# Timing and synchrony of migration in a freshwater fish: Consequences for survival

**DOI:** 10.1111/1365-2656.13790

**Published:** 2022-08-07

**Authors:** Kaj Hulthén, Ben B. Chapman, P. Anders Nilsson, Lars‐Anders Hansson, Christian Skov, Jakob Brodersen, Christer Brönmark

**Affiliations:** ^1^ Department of Biology—Aquatic Ecology Lund University Lund Sweden; ^2^ Division of Evolution and Genomics, School of Biological Sciences University of Manchester Manchester UK; ^3^ National Institute of Aquatic Resources Technical University of Denmark (DTU) Silkeborg Denmark; ^4^ Department of Fish Ecology and Evolution Center for Ecology, Evolution and Biogeochemistry EAWAG Swiss Federal Institute of Aquatic Science and Technology Kastanienbaum Switzerland; ^5^ Department of Aquatic Ecology & Evolution Institute of Ecology and Evolution, University of Bern Bern Switzerland

**Keywords:** aquatic ecology, individual differences, migration, movement ecology, predation risk, timing

## Abstract

1. Animal migration is one of the most spectacular and visible behavioural phenomena in nature with profound implications for a range of ecological and evolutionary processes. Successful migration hinges on the ability to exploit temporary resources (e.g. food) and evade threats (e.g. predators) as they arise, and thus the timing of migration is often regarded as a dominant predictor of individual migratory success.
2. However, with the exception of intensively studied taxa (mainly birds), relatively few studies have investigated inter‐individual annual and seasonal variation in migratory timing and performance, or tested predictions on how migration across high and low predation‐risk habitats may exert selection on migratory timing. In particular, studies that assess the survival consequences of variation in migratory timing remain rare, which is most likely due to the logistical challenges associated with monitoring survival success and population‐level characteristics simultaneously.
3. Here, we address the above‐mentioned questions using roach *Rutilus rutilus*, a fish that migrates from lakes characterised by high predation risk into low‐risk streams during winter. Specifically, we used individual‐based tracking of roach in two European lake systems over multiple migration periods (9 and 7 years respectively), to obtain highly detailed (year‐round scheduling, repeat journeys and the fate of individuals) data on the variability/synchrony of migratory timing in spring and autumn respectively.
4. We report seasonal differences in the variability of migratory timing, with lower variance and higher migration synchrony in spring lake arrival timing as compared to autumn lake departure timing. Furthermore, the timing of autumn migration is more variable across years than the timing of spring migration. Second, we find that later arrival to the lake habitat is positively associated with apparent survival from 1 year to the next, whereas we found no effect of lake departure timing on survival probability.
5. These findings represent rare evidence showing how intraspecific variation in timing in migratory fish differs across years and seasons, and how variation in timing can translate into survival consequences for prey in systems characterised by high predation risk.

## Graphical abstract

Timing is key for migratory success. This study investigated annual and seasonal variation in migratory timing over multiple seasons in a freshwater fish. The authors documented differences in variance and migration synchrony across seasons and show that later arrival timing to the summer lake habitat is linked to higher survival.

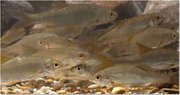

## 1 INTRODUCTION

The movement of individuals is a fundamental, ubiquitous and extremely diverse feature of the animal world, with implications for disparate ecological and evolutionary processes (Bauer & Hoye, 2014; Fudickar et al., 2021; Nathan et al., 2008). Animal migration, the movement between well‐defined habitats on a temporally predictable basis ranks among the most visible and impressive movement phenomena in nature and has evolved repeatedly in a diverse array of species as a behavioural response to cope with spatio‐temporal environmental heterogeneity in resources and/or risks (Dingle, 2014; Fryxell & Sinclair, 1988; Hansson & Åkesson, 2014). Hence, by adopting a migratory lifestyle, animals are able to effectively exploit seasonal habitats with high‐quality resources and/or a beneficial climate, as well as escaping from natural predators, parasites and severe weather conditions (Altizer et al., 2011; Dingle, 2014; Skov et al., 2013). In seasonally fluctuating environments, successful migration thus hinges on the ability to exploit temporary resources and/or avoid threats as they arise (Brönmark et al., 2008; Dermond et al., 2019; Sabal et al., 2021). Hence, the timing of migration, which directly determines the degree of spatio‐temporal overlap migratory animals experience with temporal peaks in key resources, opportunities and/or risks, is often regarded the all‐dominant predictor of migratory success (Bauer et al., 2016; Gienapp & Bregnballe, 2012; McNamara et al., 2011). Migratory timing is often the result of trade‐offs between multiple and variable selection pressures, whose strengths may vary at different stages in the migratory cycle. It is widely reported that pre‐breeding (spring) migratory timing often shows a reduced variance and appears to be more constrained and synchronised among individuals as compared with the timing of the postbreeding (autumn) migration (Alerstam et al., 2006; Cagnacci et al., 2011; Nilsson et al., 2013). The apparent stronger time‐associated selection leading to more synchronised migration in spring is often assumed to derive from the fitness benefits associated with early arrival at the breeding grounds, which has been shown to enhance breeding success (Newton, 2008; Smith & Moore, 2005). However, deviations from the population norm with regards to migratory timing, including arriving too early, can also incur substantial challenges and costs, yet how migratory timing affects key components of fitness, such as survival from 1 year to the next, has received comparatively less attention (Lerche‐Jorgensen et al., 2018).

For most animals, predation constitutes a primary source of mortality, and while historically relatively neglected in migration research, it is now increasingly recognised as an important factor in shaping migratory dynamics across taxa as diverse as zooplankton, fish and birds (Brönmark et al., 2008; Chapman, Brönmark, et al., 2011; McKinnon et al., 2010; Sabal et al., 2021; Sha et al., 2021). The risk of predation may rapidly change when migratory prey animals traverse across heterogeneous landscapes (Sabal et al., 2021). This is partly because individual predation risk depends on the distribution and density of potential predators, which often vary across the habitats migrants experience during their life cycle (Brönmark et al., 2008, 2014), but also because migration can lead to major seasonal shifts in the density of the overall prey community (Hansen et al., 2020), and, thus, also shifts in prey per‐capita predation risk (Turner & Pitcher, 1986). Theoretical models have been developed to explicitly explore the mechanisms by which frequency‐dependent predation risk can exert selection on migratory timing (Harts et al., 2016). Because the per capita risk of predation often is inversely related to population size, migrants in the wintering grounds benefit from a numerical dilution of predation risk due to a higher total abundance of individuals, as compared to spring breeding grounds. Hence, the first individuals that arrive at the breeding grounds will constitute a relatively large proportion of the local prey community. Directional movements of individuals from one destination (wintering grounds) to another (breeding grounds) will sequentially ‘dilute’ the risk for any given individual, and the degree to which predation risk is diluted depends on prey arrival time order (Harts et al., 2016). This model thus predicts that, because of risk‐dilution effects, predation risk should select for later and more synchronous spring arrival of migratory prey. However, despite the theoretical value of the ‘safety in numbers’ effect and the protective properties of grouping with conspecific migrants, few empirical studies have derived and subsequently tested predictions regarding timing and survival success in prey migrating in high predation‐risk landscapes (Furey et al., 2016).

Freshwater fishes have emerged as important models and an excellent empirical substrate to study the causes and consequences of animal migration (Brönmark et al., 2014; Chapman, Hulthén, et al., 2012; Lucas & Baras, 2008). Contemporary studies, largely focused on breeding migrations in disparate species of fish, have provided evidence that individuals are consistent in their migratory timing and that timing may be associated with fitness components such as growth, age at maturity as well as survival (Birnie‐Gauvin et al., 2021; Jensen et al., 2020; Tibblin et al., 2016). Our model organism, the roach *Rutilus rutilus* L., is a widely distributed freshwater fish and a seasonal migrant in many temperate European lakes (Borcherding et al., 2002; Hansson et al., 2007; Jepsen & Berg, 2002). Many populations of roach are partially migratory; some individuals are resident year‐round, whereas others undertake short‐distance (rarely more than a few kilometres) seasonal migrations into connected streams over winter (Brodersen, Nilsson, et al., 2008; Chapman, Skov, et al., 2012; Skov et al., 2010). Multiple studies have revealed that a large proportion (26%–73%; between‐year variation) of the roach population can reside in the streams at the same time, although the cumulative percentage of the roach population that engage in migratory behaviour can be higher (Brodersen, Nilsson, et al., 2008; Hansson et al., 2007; Skov et al., 2019). Earlier studies have shown that the seasonal migration in roach is driven by habitat‐specific changes in a trade‐off between predation risk and growth potential (Brönmark et al., 2008). The lake habitat is characterised by a high risk of predator‐induced mortality due to high densities of pike *Esox lucius* and piscivorous birds *Phalacrocorax carbo* spp., which are the principal predators of roach in these systems (Nilsson, 2006; Skov et al., 2014), but lakes also contain abundant zooplankton, peaking during spring (Hansson et al., 2007). The streams connected to the lakes generally hold low densities of predators (few stationary predators, and predators do no follow migratory prey into the streams), but streams have a comparably low food supply year‐round (Brodersen, Nilsson, et al., 2008; Chapman et al., 2013). The trade‐off between predation risk and growth potential changes in the lake with seasonally changing temperatures and, hereby, in order to maximise growth potential while minimising predation risk (i.e. to maximise fitness), roach should migrate out of the lake during winter and return to the lake in spring (Brönmark et al., 2008). This seasonal change in the trade‐off between predation risk and growth potential predicts the timing of migration at the population level (Brönmark et al., 2008). Empirical evidence also supports the axioms of the trade‐off model as migrants benefit from a reduced predation risk in the streams during winter (Skov et al., 2013), while paying a foraging cost by migrating to a relatively food‐poor habitat (Chapman et al., 2013). Moreover, migration can be facultatively induced by experimental manipulations to food availability and perceived predation risk (Brodersen, Nilsson, et al., 2008; Hulthén et al., 2015).

Here, we used individual‐based tracking of roach in two European lakes over multiple migration periods (9 and 7 years respectively), to obtain highly detailed (year‐round scheduling, repeat journeys and the fate of individuals) data on the variability/synchrony of the migratory timing in spring and autumn. The inclusion of two different lake systems allowed us to explore if observed patterns were shared across independent migratory populations. We predicted that migration into the lakes (high‐risk habitat) in spring should be more constrained and synchronous among individuals as compared to autumn migration out of the lakes into the streams (low‐risk habitat) as frequency‐dependent predation risk should exert stronger selection pressure in spring as compared to autumn. This variation should be mirrored both at the population level (lower year‐to‐year variation in average lake arrival dates) and at the level of individuals tracked during multiple years (less pronounced year‐to‐year flexibility in individual lake arrival dates). Similarly, if selection for high migration synchrony is more important in spring than *autumn, and migrants returning to the lake with depleted energy reserves are experiencing* frequency‐dependent predation risk, we predict that survival should be strongly linked with the timing of spring migration, but not necessarily to autumn migration. Specifically, we predicted early spring migrants to experience mortality costs, as fish migrating (too) early would benefit less from numerical risk dilution when arriving to the lake summer habitat. Similarly, we predicted a stronger selection for high arrival synchrony at the inter‐individual level (within years) during spring migration as increased densities of co‐migrants could act to swamp predators (Furey et al., 2016) and thus reduce roach per capita risk. Finally, as predator–prey interactions are inherently size dependent, for example due to gape limitation of predators (Skov et al., 2011), we predicted larger fish to demonstrate higher survival rates.

## 2 MATERIALS AND METHODS

### 2.1 Study systems

We studied the seasonal migration of roach using data from individually based, long‐term monitoring programs in two European lakes, Krankesjön and Søgård Sø. Krankesjön, (55°42′N, 13°28′E) is a small, shallow (2.9 km^2^; average depth 1.5 m) and moderately eutrophic lake in southern Sweden (Hargeby et al., 1994). Large parts of the lake are covered with dense stands of submerged vegetation (mainly *Chara* spp.) and the western and south‐eastern parts are fringed by extensive beds of reed (*Phragmites australis* cav. [Hansson et al., 2013]). Søgård Sø (55°25′N, 9°19′E) is a small, shallow (0.26 km^2^; average depth 1.6 m) and eutrophic lake in southern Jutland, Denmark. The lake has no submerged vegetation, but a 3–4 m wide reed margin (Grünfeld, 2003). Roach and Eurasian perch *Perca fluviatilis* L. numerically dominate the fish assemblage in both lakes, and pike *Esox lucius* L., larger size classes of perch, and cormorants *Phalacrocorax carbo* L. are the principle predators of roach (Grünfeld, 2003; Skov et al., 2008). Previous studies have shown that a substantial fraction (up to 80%) of the roach populations in these lakes migrates seasonally from the lake summer habitat into streams connected to the lakes (Krankesjön: one outlet and two inlet streams, Søgård Sø: one inlet and one outlet stream).

### 2.2 Sampling and tagging of fish

We caught roach each year, from 2003 to 2011 in Krankesjön and from 2005 to 2011 in Søgård Sø, by electrofishing and beach seining from early September to late October (with the exception of 2003 when fish were sampled until late November in Krankesjön). Following capture, we measured the total length (nearest mm) of all individuals. Fish were then anaesthetised (benzocaine) and individually tagged by surgically implanting a uniquely coded TIRIS Passive Integrated Transponder (PIT) tag (Texas Instruments, RI‐TRP‐RRHP, half duplex, 134 kHz, 23.1‐mm long, 3.85‐mm diameter, 0.6 g in air) into the body cavity. This method of PIT tagging has previously been evaluated and has no observable effects on survival or body condition in roach (Hulthén et al., 2014; Skov et al., 2005, 2020). In total, we tagged 4093 individuals in Krankesjön (on average 455 individuals per year; range: 96–694 individuals; total length 151 ± 25 mm) and 1909 individuals in Søgård Sø (on average 273 individuals per year, range 205–492 individuals; total length 159 ± 24 mm). After tagging, fish were released back into their lakes of origin at the approximate location of capture. The study complies with the current laws in Sweden and Denmark; ethical concerns on care and use of experimental animals were followed under permission (M165‐07) from the Malmö/Lund Ethical Committee and the guidelines described in the permission (2012‐DY‐2934‐00007) from the Danish Experimental Animal Committee.

### 2.3 Field monitoring of fish migration

We monitored the migratory patterns of individual roach between lakes and connected streams using passive biotelemetry with stationary, continuously operating antenna arrays (Castro‐Santos et al., 1996; Skov et al., 2008). Two loop‐shaped antennas, each covering the entire cross‐section of the streams, were placed 3–6 m apart. When a tagged fish swims through, or in the vicinity of, an antenna, the tag is energised and emits a unique identity code that is stored with a date and time stamp on a memory card on a RFID multiplexer unit. The use of paired antennas in each stream enables the determination of fish swimming direction based on the sequence of detections. The recording frequency was set to five energise/receive cycles per second. PIT‐tag monitoring systems were installed in 2003 in Krankesjön and in 2005 in Søgård Sø. Thus, we have data from 10 and 8 consecutive years of monitoring of individual migrants in Krankesjön and Søgård Sø respectively. Studies employing PIT tags to monitor fish behaviour and survival may be subject to imperfect detection efficiency. However, a calibration exercise on the PIT‐tag antennae system we use in this study has shown that the likelihood of a tag not being recorded was extremely low (<0.2%: Chapman, Brönmark, et al., 2011; Chapman, Hulthén, et al., 2011). During each season, migration was monitored between 1 August, before autumn migration, and 1 July, when spring return migration to the lake had ended (except for the first season 2003/2004 in Krankesjön when monitoring started in October due to late installation of antennae). We only included the migration patterns of individual fish with a minimum of 5 days between the first and last record to exclude erratic movements in the vicinity of an antenna. Throughout, the timing of lake departure and arrival was defined as the first and last time an antenna registered a given individual within a migration period.

## 3 DATA TREATMENT AND ANALYSIS

We tested for between‐year variability in lake departure and arrival dates at the population level by calculating the average timing of migration from the lake to the stream habitat, and vice versa, for each year and lake. Electronic tagging benefits from being performed at relatively low water temperatures (Skov et al., 2005), and, hence, the first few migratory individuals could potentially have initiated their winter migration already before tagging. In order to obtain data unbiased of potential tagging stress and by missing out on early migrants we therefore calculated lake departure data from individuals migrating the season after they were initially tagged (Brodersen, Nicolle, et al., 2011). On average, we used 65 (total *n* = 580) and 48 (total *n* = 336) individuals per year to estimate the mean annual date of lake departure in lakes Krankesjön and Søgård Sø respectively. In our study lakes, migration occurs over relatively short distances and some individuals may switch between the lake and stream habitats several times during a year. Because mortality during short lake visits could lead to a bias towards earlier arrival, our estimates of the average timing of migration from the stream to the lake habitat are based on arrival data from fish being last positioned at the antenna closest to the lake (i.e. controlling for in‐stream mortality) and on individuals also registered the year after data were collected (i.e. individuals that we knew for certain were alive during the whole preceding migration period). On average, we used 55 (total *n* = 492) and 52 (total *n* = 362) individuals per year to estimate the annual date of lake arrival in Krankesjön and Søgård Sø respectively. To compute descriptive statistics (circular standard deviation, hereafter CSD) and to visualise inter‐annual variation in lake departure and arrival timing at the population level, we converted migration dates (day of the year) into angles and treated migration timing as a circular variable (Batschelet, 1981; Zar, 2019). In this way, the year is represented on the circumference of a circle with a phase of 365 with day 1 (1 January) at 0 radiant (Cagnacci et al., 2011).

We also explored year‐to‐year variability in the timing of autumn and spring migration at the level of individuals. To achieve this, we exploited data originating from fish individuals that were tracked for multiple and consecutive years (from three to six consecutive years). Individual‐level timing data were pooled across years and, following the same rationale as above, we excluded data for departure timing the same year as tagging occurred and for arrival timing the last year a given individual was monitored. The total dataset thus consisted of individual timing data for 1318 departures and arrivals, made by 171 and 111 repeatedly tracked individuals in Krankesjön and Søgård Sø respectively. For each individual, we computed the intra‐individual circular standard deviation for lake departure and arrival timing. We then tested for differences in CSD between seasons for repeated journeys made by the same individuals with conservative paired‐sample sign test since the paired differences were neither normally nor symmetrically distributed. To assess the degree of migration synchrony among individuals across seasons, we used circular statistics to compute the length of the mean vector r (circular measure of dispersion) for departure and arrival data for each lake and year. The length of the r vector ranges from 0 to 1 and is inversely proportional to the standard deviation of migration dates across individuals. Larger values thus indicate a greater clustering around the mean, and, hence, a higher degree of migration synchrony among individuals (Cagnacci et al., 2011, 2016). The r‐vector values were compared between seasons with a *t*‐test for Krankesjön data and a Mann–Whitney *U* test (due to non‐normality) for Søgård Sø data. Next, we analysed the effect of the timing of autumn and spring migration on the survival of roach. In these analyses, we took advantage of the high individual consistency in migratory tactic (i.e. residency/migration) in this species (Brodersen et al., 2014). For example, of the 294 individuals that during a period of three or more years after their initial migration migrated again, 97.8% (178 of 182) and 99.1% (111 of 112) did so during consecutive years in Krankesjön and Søgård Sø respectively. Hence, extremely few individuals shifted their migratory tactic between years (i.e. switched from migration to residency and back again), which strongly implies that cases where an individual migrated in year_x_ but not in year_x+1_ is most likely due to mortality rather than plasticity in the decision to migrate or remain resident. Hence, return migration from 1 year to the next should be a good proxy for survival. Again, to control for mortality in the stream we only included migrants last being positioned at the antenna closest to the lake, within a given migration season. This resulted in a dataset with 1,393 and 823 individuals in lakes Krankesjön and Søgård Sø respectively. We determined the relative migration date (Vardanis et al., 2011) expressed as the difference between the average migration date of all migrants (within a given year, season and lake) and the actual timing of survivors and non‐survivors, respectively and no individuals were tracked during multiple years in the survival analysis. In this way, positive scores indicate later migration (relative to the population mean), whereas negative scores indicate earlier migration. Subsequently, logistic regression models (likelihood ratio backwards elimination with selection criteria at *α* = 0.05) were fitted to model the binary outcome of survival (yes/no) as a function of the independent factors relative lake departure or arrival date, body size at tagging (TL), as well as their interaction term (see Table S1 for an output of the stepwise backward elimination process). We subsequently visualised survival patterns in relation to timing as lake and year‐specific deviations from the population mean for survivors and non‐survivors respectively.

## 4 RESULTS

**FIGURE 1:**
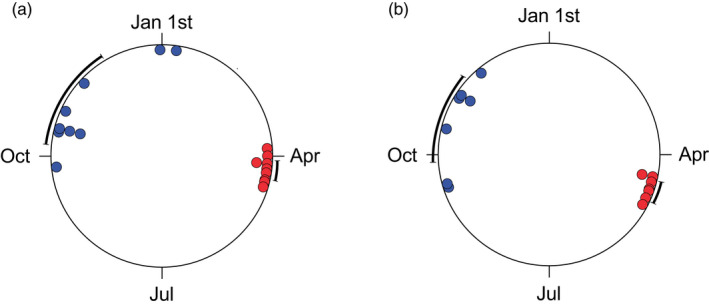
Temporal migration patterns in roach in the two study lakes, lake Krankesjön, Sweden (a), and lake Søgård, Denmark (b). The large circle represents the year and small solid circles on the perimeter represent the average timing of migration of the population for a specific year. Blue circles represent autumn migration timing and red circles spring lake arrival timing. Bold error bars outside the large circle denote 95% CI of autumn and spring migratory timing across years respectively.

**FIGURE 2:**
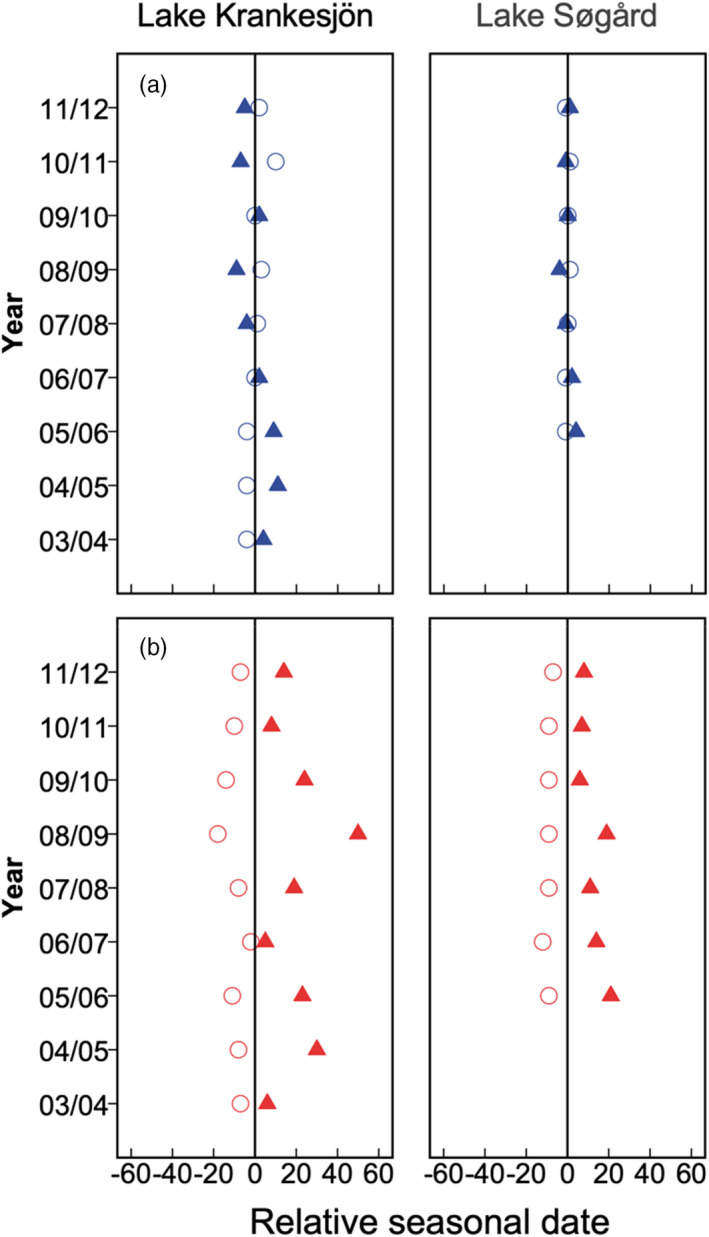
Relative timing of roach autumn (a; blue symbols) and spring migration (b; red symbols) in lake Krankesjön (left panel) and lake Søgård (right panel). The solid vertical line denotes the average timing of migration for all roach individuals within a specific year and the symbols represent the relative deviation from the average for survivors (filled triangles) and non‐survivors (open circles) within a given migration season (*y*‐axis). In autumn, survivors and non‐survivors do not differ in migratory timing, whereas in spring survivors migrate back to the lake later than non‐survivors.

Across years, the average autumn migration from the lake to the wintering habitat in the streams occurred on 4 November and 20 October, while return migration in spring occurred on average on 8 and 20 April, in lake Krankesjön and Søgård Sø respectively. The timing of autumn migration was considerably more variable across years than the timing of the spring migration (Figure 1; see Figure S1 for circular frequency histograms of individual departure and arrival dates). In Krankesjön, the average autumn lake departure for a specific year occurred between 25 September and 9 January (range: 106 days, CSD = 34.2) in the nine consecutive years of migration monitoring, whereas average spring lake arrivals were constrained to a period of 21 days (29 March–19 April, CSD = 6.4, Figure 1a). In Søgård Sø, average autumn lake departures spanned over 70 days (12 September–21 November CSD = 24.6) over the 7 years of monitoring, whereas spring lake arrivals were constrained to a period of 17 days (14 April–1 May, CSD = 6.0, Figure 2b). Our analyses also revealed seasonal differences in the variability of migratory timing within the same individuals tracked across consecutive years of migration. The variation in intra‐individual timing of the autumn migration was greater than for the spring migration, a pattern that was shared between lakes (Sign test, Krankesjön: *Z* = −6.442, *p* < 0.001, Søgård Sø: *Z* = −4.981, *p* < 0.001). We also found that spring arrival timing to the lake habitat was more synchronised among individuals, as quantified by mean vector lengths (*r*), as compared to the autumn lake departure, a pattern also shared across lakes (Krankesjön spring arrival: mean *r* = 0.924, autumn departure: mean *r* = 0.743; *t*‐test, *p* = 0.0002; Søgård Sø spring arrival: mean *r* = 0.981, autumn departure: mean *r* = 0.690, Mann–Whitney *U* test, *p* = 0.003). With regards to the association between lake departure timing and survival, logistic regression with backwards elimination revealed that neither fish body size (Wald = 0.343; *p* = 0.558), nor the body size × departure timing interaction term (Wald = 1.695; *p* = 0.193) or lake departure timing (Wald = 1.299; *p* = 0.254) affected the probability of survival from one season to the next. Similarly, we found no effects of body size (Wald = 0.013; *p* = 0.909) or the body size × arrival timing interaction term (Wald = 0.321; *p* = 0.571) on survival. However, lake arrival timing was found to strongly affect the probability of survival (Wald = 122.378; *p* ≤ 0.001, *β* = 0.017 ± 0.0029). Hence, survivors arrived to the lake habitat later as compared to the population average, whereas non‐survivors arrived earlier, indicating a survival cost of early arrival (Figure 2).

## 5 DISCUSSION

Using long‐term data on individual migration patterns in roach, we show that there are strong seasonal differences in the variability of migratory timing, with lower variance and higher migration synchrony among individuals in spring lake arrival timing as compared to autumn lake departure timing. This pattern is robust across study lakes and across years, and also within individuals migrating over multiple years. Our data fit well with migration theory, which predicts that different selection pressures and variation in the strength of time constraints act upon migrants during different periods in the annual cycle (Kokko, 1999; Newton, 2008; Nilsson et al., 2013). In general, spring (pre‐breeding) migration is regarded as more predictable and time‐selected and as compared to autumn (postbreeding) migration (Åkesson et al., 2017; Conklin et al., 2013; Kokko, 1999; McNamara et al., 1998). However, much of the current data examining seasonal variation in migratory timing and performance is drawn from empirical studies of birds (Alerstam et al., 2006; Nilsson et al., 2013; Stanley et al., 2012; Vardanis et al., 2011). In avian migrants, selection for early arrival and increased synchrony in the timing of spring arrival is primarily related to the peak of the spring resource base and to reproductive opportunities where prior residency gives a competitive advantage for the best territories that have higher resource levels and higher probabilities of attracting mates, and hence increases breeding success (Kokko, 1999; Smith & Moore, 2005). There is also empirical evidence in support of increased synchrony in pre‐breeding (spring) migration timing in large mammals, such as ungulates (Cagnacci et al., 2011; Monteith et al., 2011).

If the fitness costs of suboptimal migration timing are higher in spring than autumn, selection should act to reduce variation and increase synchrony in migratory timing in a more pronounced way in spring. While this hypothesis is intuitive, empirical data in support of seasonal differences in fitness costs of migratory timing are rare. Fitness costs can be expressed in different ways, for example via a reduced reproductive output, or more immediately via a survival cost to ‘mistiming’ the migratory journey. We tested the hypothesis that the ultimate driver of the seasonal variation in migratory timing that we documented in roach is a mortality cost to missing an optimal time of migration in spring, but not autumn. We found that survival was strongly linked with the timing of lake arrival, whereas this was not the case for lake departure timing. A relatively early return migration to the lake habitat decreased the likelihood of migration during the following season, a pattern that was consistent across years and shared across study lakes.

From an adaptive perspective, early arrival has been puzzling, and the costs of early arrival remain much less acknowledged than its benefits (Lerche‐Jorgensen et al., 2018). However, Harts et al., 2016 gave later arrival an adaptive explanation by showing that frequency‐dependent predation risk can select not only for later arrival, but also for more synchronous arrival. Experimental systems such as ours, characterised by high risk and in which predators play a key role for migratory dynamics, via, for example, influencing individual migration propensity, timing and survival (Brönmark et al., 2008; Hulthén et al., 2015; Skov et al., 2011, 2013) offer an opportunity to empirically test survival consequences of migratory timing relative to other individuals within a given population. First, our finding of survival benefits associated with late arrival aligns well with theoretical considerations on how individual predation risk may rapidly change when migrants traverse across habitats (Harts et al., 2016). The first roach individuals that migrate from the stream to the lake will constitute a relatively large proportion of the potential prey community in the high predation‐risk lake habitat. Directional movements of individuals from stream wintering grounds to lake summer grounds will sequentially ‘dilute’ the risk for any given individual, and the degree to which predation danger is diluted depends on arrival time order. Hence, individuals that arrive relatively late to the lake, when many conspecific migrants have already returned and are residing in the lake habitat, should benefit more from a predator dilution effect and are thus predicted to demonstrate higher survival rates, which is also what we report. This, however, does not explain why some individuals arrive earlier than the bulk of the population. As this also occurs for many individuals that have previously migrated and successfully returned, a lack of individual experience is unlikely to be the primary explanatory factor. Previous experimental work has revealed that individual condition is linked to the timing of migration out of the lake and also back to the lake in spring. Roach in good condition migrate earlier to the streams in the autumn, whereas roach in poor condition return early to the lake in spring (Brodersen, Nilsson, et al., 2008). It is hence plausible that individual differences in timing reflect underlying differences in energy accumulation performance prior to migration, as well as differences in metabolic rates. Individuals with low energy stores and/or high metabolism may be able to survive the winter in the stream when temperatures and metabolism are low, but when temperature and metabolism increase in spring their energy reserves are depleted and they are, thus, forced to migrate back early and during suboptimal periods (Brodersen, Rodriguez‐Gil, et al., 2011; Brodersen, Nilsson, et al., 2008). Malnutrition and poor body condition may also induce risk‐prone behaviours (McNamara & Houston, 1987), such as compensatory feeding, in early returning individuals, which in combination with high per capita risk of predation early in the season could act as to increase individual predation susceptibility.

In the theoretical model developed by Harts et al., 2016, predator‐mediated selection was also shown to be a key driver of arrival synchrony, because individuals deviating from the population norm by arriving (too) early will pay higher predation‐risk costs whereas individuals arriving (too) late will miss out on access to key recourses (e.g. mating and foraging opportunity). Return migration to the lake habitat in spring occurs in relatively close proximity to the spawning period and the majority of tagged fish in the current study should be sexually mature. Roach have a lek‐like mating system in which females express preferences for individual males, for example those with established territories (Kortet et al., 2004; Wedekind, 1996), and, further, food is particularly abundant during the zooplankton peak during spring (Hansson et al., 2007), highlighting the potential costs of too late arrival in this species. A high degree of arrival synchrony (increased densities of co‐migrants) can also derive from selection to numerically swamp predators (Furey et al., 2016). Several studies have shown that resident predators can partially switch to and binge feed on migratory species soon after they appear (Furey et al., 2015). Migrations of roach have also been shown to affect the foraging ecology of top predators, and cross‐lake comparisons have revealed that resident predators in lakes with migration opportunity for prey show a higher degree of feast‐and‐famine as compared to predators in lakes where potential prey do not have the opportunity to migrate (Hansen et al., 2020). Hence, in study systems such as ours, key predators of roach appear to be adapted to capitalise on short pulses of food, such as the return migration of roach in spring. A high degree of arrival synchrony can thus serve to swamp the short‐term capacity of predators that may aggregate to consume roach before they disperse in the lake habitat to seek refugia and restore energy reserves.

In the autumn, seasonal changes in predation risk and growth rate change more gradually than during spring (Brönmark et al., 2008), which sets the scene for less variance and more pronounced migration synchrony in spring as compared to autumn. Similarly, the more variable autumn migration can potentially reflect year‐to‐year variability in weather, predator and resource abundance, as well as inter‐individual variation in physiological state. For example, in controlled food‐manipulation experiments, roach provided with elevated food levels migrated earlier as compared to individuals with restricted access to food prior to the migration season (Brodersen, Nilsson, et al., 2008). The cost/benefit analysis of when to migrate may thus differ across years (because of interannual variability in for example food supply) but may also be a function of *inter*‐*individual differences* in the ability to acquire the necessary resources to fuel migration, behaviour and underlying metabolic rates (Brodersen, Nilsson, et al., 2008; Chapman, Hulthén, et al., 2011). Accordingly, we show here that autumn migration has a higher degree of variability and less synchrony than the spring migration, and that autumn migration timing is less directly associated with survival.

In a broader context, the patterns we document here may have significant consequences for the ecosystem dynamics of shallow lakes since the timing of fish migrations to and from the lake may affect the population dynamics of herbivorous zooplankton (Brodersen, Nicolle, et al., 2011; Hansson et al., 2007), shaping the biomass development and succession of phytoplankton and thereby altering the potential for algal bloom formation. Moreover, in lakes undergoing major transitions between alternative stable states on a decadal time‐scale, such as lake Krankesjön (Brönmark et al., 2010), the timing of spring migrations of zooplanktivorous fish may be a powerful factor influencing the state transition of lakes (Brodersen, Ådahl, et al., 2008). However, the strong selection for synchrony in spring that we report here may act to buffer the stability of the lake ecosystem from switches between alternate stable states, highlighting the potential for animal migration to have profound ecological implications (Bauer & Hoye, 2014; Brodersen, Ådahl, et al., 2008).

## AUTHOR CONTRIBUTIONS

All authors conducted fieldwork, collected data and contributed to the design of the study; Kaj Hulthén and P. Anders Nilsson. performed the statistical analyses and prepared figures and tables; Kaj Hulthén led the writing and revisions to which all authors contributed. All authors have approved the submitted manuscript.

## CONFLICT OF INTEREST

The authors declares that there is no conflict of interest.

## Supporting information


Figure S1
Table S1

## Data Availability

Data available from the Dryad Digital Repository https://doi.org/10.5061/dryad.qz612jmjn (Hulthén et al., 2022).
