# Supplementary material for: Timing and synchrony of migration in a freshwater fish: Consequences for survival
Source: J Anim Ecol. 2022 Aug 7;91(10):2103–12. doi: 10.1111/1365-2656.13790 (PMC9805062; doi:10.1111/1365-2656.13790)
Supplement: Supplementary file 1 — Figure S1 Table S1 [file JANE-91-2103-s001.docx]

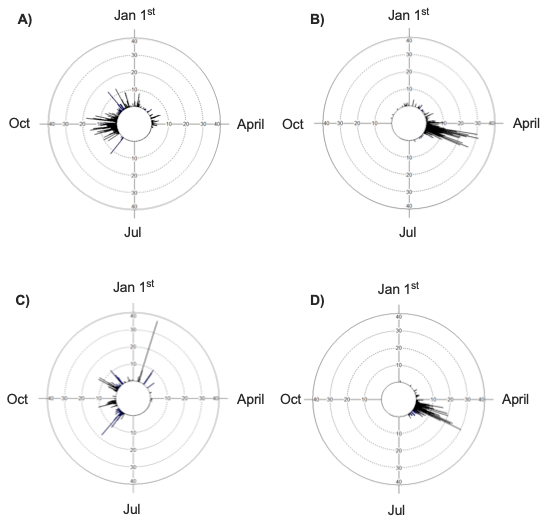


**Figure S1**. Circular histograms (the large circle represents the year) showing individual lake departure and arrival data in lake Krankesjön, Sweden (A, B) and in lake Søgård, Denmark (C, D).

**Table S1**. Output from logistic regression models (likelihood ratio backwards elimination with selection criteria at α = 0.05) fitted to model the binary outcome of survival (yes/no) as a function of the independent factors relative lake arrival (top) or departure (bottom) date, body size at tagging (TL), as well as their interaction terms
